# Stop lecturing and start teaching

**DOI:** 10.1120/jacmp.v16i6.6042

**Published:** 2015-11-08

This issue's editorial is authored by George Starkschall, former Editor of the *JACMP* and Chair of the Education Council. He is uniquely qualified to offer a perspective on how to encourage students to learn medical physics.

Michael D. Mills, PhD

Editor‐in‐Chief

The AAPM website (http://www.aapm.org/publicgeneral/default.asp) describes the roles of the medical physicist as follows:

"Medical physicists are concerned with three areas of activity: clinical service and consultation, research and development, and teaching. On the average their time is distributed equally among these three areas."

Whether this distribution of effort is correct may be subject to debate, but at least a significant portion of the workload of many medical physicists is spent in education. Especially for medical physicists in an academic setting, much of this time is spent in formal courses — teaching medical physics students, medical residents, or allied health personnel such as radiography students and radiation therapy technology students.

Given that a medical physicist's responsibilities often include formal teaching, I want to challenge medical physicists who are involved in formal teaching with two questions:

- Question 1: How are your present medical physics practice methodologies different from those you used five years ago?
- Question 2: How are your present teaching methodologies different from those you used five years ago?

The first question is an easy one to answer, given the many changes that have occurred in our field over the past five years. The second, however, is more difficult to answer. Some medical physicists may update their lecture notes from year to year, but for many, the course content does not change, nor do the notes. After all, not much has changed in the Compton effect as it relates to medical physics since the derivation of the Klein‐Nishina formula in 1928.^(1)^ I recall when I was a graduate student, one of my physics professors, a distinguished Nobel laureate, lectured from the same worn notebook he had used for at least 25 years, if not more.

The fact is that the primary purpose of our lectures is to transmit information to our students. We medical physicists typically do not know very much about the actual process of learning. We often patterned our behavior after effective teachers we had in college or graduate school. We did not have the opportunity to learn the theory of learning, so we learned by example. In that respect, we are educational technologists; we know how to do, but we do not necessarily understand the why of what we do.

The standard teaching methodology we learned is to present information in a 45‐ to 60‐minute lecture. This approach to teaching is a one‐way, instructor‐focused approach. The instructor stands at the front of the lecture hall and is the center of attention, distributing information to the assembled listeners.

One size fits all. Information is dispensed at a pace determine by the instructor, with little, if any, real‐time feedback. Little attempt is made to assess whether or not the student is absorbing the information. If the student loses the train of thought (e.g., falls asleep), it may be hard for the student to catch up and much of the lecture content is lost.

The formal lecture is also an inefficient use of the lecturer's time. If the content of the lecture does not change significantly from that of previous years, why not use last year's lecture? If another lecturer may do a better job of presenting the information, why not use one of that lecturer's canned lectures? The instructor's class time can be better spent clarifying concepts than presenting information. "In the digital world, there is no longer any reason to use class time to transfer the notes of the instructor to the notes of the student (without passing through the brain of either)."^(2)^

One method for making more efficient use of the lecturer's time and expertise is a technique known as "flipped learning". Unlike the traditional way of teaching, in which class time is used for presenting new materials while out‐of‐class time is used for working examples and problems, in flipped learning, new information is presented out of class, and class time is used for working examples and problems. Rather than spending valuable classroom contact time in presenting information via a lecture, the teacher can record the lecture, post it on a website, and allow students to listen to the lecture at their leisure.

Several points in incorporating a flipped learning model in the physics classroom are well worth noting. It is important to verify that students have prepared for class by listening to the recorded lecture. Verification can be demonstrated in several ways. In my classes, I give the students a short online quiz prior to class. This quiz has to be completed the morning of the class day. The quiz typically consists of five relatively easy short‐answer questions and is ungraded. Students receive credit simply for doing the quiz. The software I use to display the quiz generates summary statistics so I can see how well the students answered the questions. If students appeared to have difficulty with a question, it becomes a topic for discussion during class.

The final question on the pre‐class quiz is the free‐form question: "Are there any issues that are not clear to you at this time?"

I compile all the questions that students have asked and start the class by answering these questions. In some years, I have used these questions to modify lectures for classes in subsequent years. The remainder of the class time is used to teach using the technique of peer instruction.^(3)^ I find that this technique ensures a great deal of active student participation. Students must actively participate in the classroom discussion and are not allowed to be passive bystanders. Moreover, through the use of an online forum, such as Facebook or a bulletin board server, classroom discussions can be continued beyond the time restrictions of the formal lecture period.

Perhaps the most difficult issue facing the potential adopter of a flipped learning teaching style is that of recording lectures to place on a website. Most people typically find it easier to stand in front of a class and lecture to an audience than to talk into a microphone. The solution I have found is to record the classroom lectures, and then have the lecture recording transcribed. A not‐too‐surprising revelation upon reading a transcription of a recorded lecture is how incoherent a speaker is when talking spontaneously. However, once the lecture has been transcribed, the transcription can be reviewed and edited, then re‐recorded from a script based on the transcription. Commercially‐available software can add a sound track to a presentation and generate an appropriate file that can be downloaded by the student from a website.

Implementation of the techniques that have been shown are likely to take medical physics teachers out of their comfort zones, but the way we learn is by extending the boundaries of our comfort zones. We venture out of our comfort zone any time we implement a new technology in the clinic; in an analogous manner we need to venture out of our comfort zone when we implement a new methodology in our teaching.

We need to remember that our role as medical physics teachers is not to teach our students medical physics. Our role as medical physics teachers is to teach our students to learn medical physics.
